# Single-Cell Endoscopy for Multifunctional Live-Cell Molecular Analysis

**DOI:** 10.3390/bios15040244

**Published:** 2025-04-11

**Authors:** Haoze Xue, Li Wang, Han Yao, Shuwei Shen, Xu Zhao, Chenxi Yuan, Luting Yu, Guoguang Chen, Jia Liu

**Affiliations:** School of Pharmaceutical Sciences, Nanjing Tech University, Nanjing 211816, China; 15940128688@163.com (H.X.); 202461109042@njtech.edu.cn (L.W.); 202361218206@njtech.edu.cn (H.Y.); suviashen@163.com (S.S.); 202221009028@njtech.edu.cn (X.Z.); 18679882777@163.com (C.Y.); ggchen@njtech.edu.cn (G.C.)

**Keywords:** single-cell analysis, live-cell technique, molecular analyses, high resolution, single-molecule sensitivity

## Abstract

Molecular analyses of individual cells with high resolution, specificity, and sensitivity can not only reveal cellular heterogeneity but also provide a better understanding of diseases and accelerate drug discoveries. Single-cell endoscopy is an advanced live-cell technique that relies on a smart endoscope that allows minimally invasive probing of the interiors of individual cells. Compared with other single-cell analysis techniques, single-cell endoscopy has shown great promise in applications such as flexible single-cell manipulation, ultrasensitive sensing, and precise intracellular delivery. In this review, we aim to map out the landscape of recent advances in single-cell endoscopy techniques by focusing on both fundamental considerations and significant progress over the past decade. Specifically, we summarize the predominant live-cell endoscopes, including their fabrication and characterization. Furthermore, a series of valuable intracellular molecular sensing events, such as nucleic acids, proteins, ions, etc., are introduced with a main emphasis on how single-cell endoscopy can solve these issues and what merits single-cell endoscopy can provide. Finally, we briefly outline the remaining challenges and directions for the future development of single-cell endoscopy techniques.

## 1. Introduction

Molecular analyses of individual cells are crucial for gaining a fundamental understanding of the molecular behaviors inside cells, which can provide valuable information for elucidating the biological processes of various diseases, including cancers, facilitating the design of new therapies, and accelerating drug discoveries [1]. Despite their convenience and efficacy in single cells, molecular analyses present several challenges in achieving high spatial resolution in single living cells for uncovering spatial heterogeneity, high sensitivity for endogenous biomolecule detection, and fine specificity in the complicated cellular environment [2].

Currently, numerous techniques have been developed for molecular analyses in single cells, including single-cell mass spectrometry [3,4], microfluidics [5,6], flow cytometry [7], single-cell Western blotting [8,9], and single-molecule imaging [10]. However, the overwhelming majority of the above techniques for highly expressed molecules tend to be limited by low sensitivity, while quantities of important biomolecules in cells are present in low copy numbers [11]. Additionally, some of the mentioned approaches, such as microfluidics and fluorescence-based imaging strategies, are destructive, for they could lyse the cells or introduce fluorescent reporters into living cells, either killing cells or perturbing the native composition of the cells.

**Figure 1:**
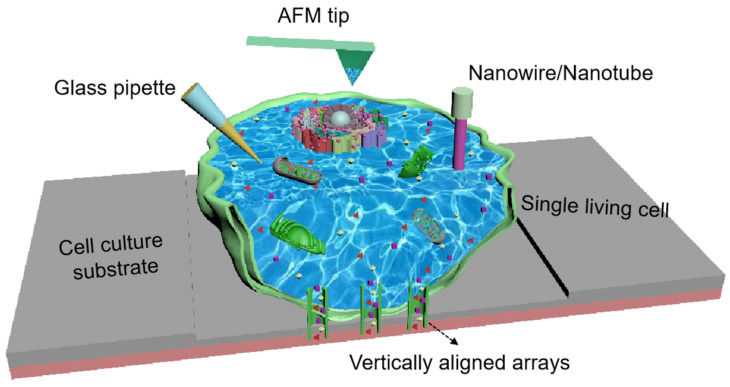
Schematic of molecular analyses in single living cells by single-cell endoscopy techniques, including glass pipettes, AFM tips, nanowires/nanotubes, and vertically aligned arrays.

To overcome these limitations, the use of smart endoscopes has been widely explored, such as glass micro-/nanopipettes [12,13,14], atomic force microscope (AFM) tips [15,16,17], or nanowires/nanotubes [18,19]. These smart endoscopes can be precisely inserted into a single living cell for multifunctional live-cell interrogation (Figure 1). Compared with other single-cell techniques, the single-cell endoscopy technique has several obvious advantages: First, it has a high resolution, which can be used to probe the intracellular environment with a spatial resolution of ~100 nm without disrupting the cell [18]. In addition, if integrated with some advanced detection techniques, such as Raman spectroscopy, single-cell endoscopy-based approaches are capable of providing single-molecule sensitivity, with a lower detection limit of ~6 target protein molecules in a single living cell [20]. Furthermore, as a live-cell technique, the nanometer-sized endoscope can be precisely inserted into cells for probing; this process is nondestructive with >95% cell viability [21]. This characteristic is of great value in academic realms, such as developmental biology and reproductive medicine.

In this review, we critically survey recent advances in single-cell endoscopy techniques, summarizing the predominant endoscopes, including their fabrication and characterization. We highlight a series of valuable intracellular molecular sensing events, such as nucleic acids, proteins, and ions, with a particular focus on how single-cell endoscopy can address these challenges and propose potential advantages it offers. Finally, we outline the remaining challenges and propose potential directions for future development of single-cell endoscopy techniques.

## 2. Fabrication and Characterization of Endoscopes

To effectively perform single-cell endoscopy analysis in single living cells, a sophisticated endoscope is essential. Nanoscale endoscopes offer advantages for their minimally invasive insertion into single living cells, enabling precise probing of the intracellular environment. Moreover, these small endoscopes also function as confined spaces that can enhance the electrochemical field or increase optical density, thereby proving detection efficiency [12]. Depending on their geometry and throughput, current endoscopic probes can be primarily classified into three categories: conical probes (such as glass pipettes and AFM tips), cylindrical probes (such as nanowires and carbon nanotubes), and vertically aligned arrays. The detailed information of different endoscopes is summarized in Table 1.

**Table 1:** Summary of the predominant single-cell endoscopes.

Type	Glass Pipettes	AFM Tips	Nanowires/ Nanotubes	Vertically Aligned Arrays
Dimension	micro: 0.5–5 µm nano: ~100 nm	~200–400 nm	~50–250 nm	diameter: ~150 nm height: 2–3 µm
Modification	antibody/ nanoparticle	usually hollow	polymer/ nanoparticle	usually hollow
Detection	fluorescence, Raman, electrochemical	fluorescence, ELISA, PCR	electrochemical, Raman	fluorescence, ELISA, PCR
Sensing	DNA/RNA/protein/ion/pH/gas	mRNA/protein	protein/glucose/ion/gas	mRNA/protein/ion
Advantages	assess heterogenous variants in RNA and mitochondrial DNA expression by precisely targeting organelles within cells	extract fl quantities of intracellular materials; accurately target organelles within cells	less invasive because of fine diameters and uniform geometry	high throughput
Limitations	cellular damage due to the relatively larger dimensions and low throughput	cellular damage due to larger dimensions and incapable of continuous fluid handling	long insertion time (usually 20–30 min) and lack of temporal control over the delivery process	the accuracy of sampling is challenging
Reference	[12,13,14]	[22,23,24]	[18,19,25]	[26,27,28]

### 2.1. Conical Endoscopes

**Figure 2:**
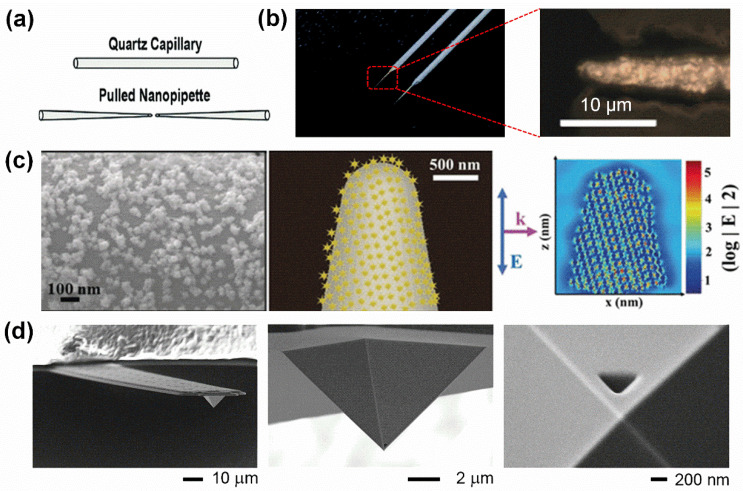
Fabrication and characterization of the conical endoscopes. (**a**) Schematic of the physical pulling route for fabrication of a starting glass pipette. Reproduced with permission [12]. Copyright 2019, John Wiley and Sons. (**b**) Representative digital photo of a starting glass micropipette with a nanometer-thick gold layer on the surface. Microscopic image of the tip of a gold-coated glass micropipette. Scale bar: 10 µm. Reproduced with permission [34,35]. Copyright 2018, Royal Society Chemistry. Copyright 2021, Springer Nature. (**c**) SEM image of a gold nanostar-modified glass nanopipette. Schematic of surface coverage and a finite difference time domain (FDTD) simulation (center, right). Reproduced with permission [33]. Copyright 2019, John Wiley and Sons. (**d**) SEM image of a FluidFM tip, which consists of a hollow cantilever with a hollow pyramidal tip (400 nm in height and base length). Reproduced with permission [36]. Copyright 2016, Elsevier.

*Glass pipettes.* Currently, glass pipettes have been used as an important endoscope because they are easy to obtain and flexible for further surface modification [13]. Typically, a starting glass pipette is fabricated from a quartz or borosilicate capillary with an inner diameter of several tens of micrometers by using a laser puller to laser heat the middle part of the capillary with a pipette puller (Figure 2a). The diameters of pulled glass pipettes are adjustable, ranging from ~100 nm to 5 µm, by regulating five editable pulling parameter settings: heat, filament, velocity, delay, and pull. Generally, a higher laser heat, force, and pulling velocity, as well as a smaller filament and delay, are more likely to obtain a sharper tip of a glass pipette [12].

Previously, the surface of a starting glass pipette was directly immobilized with antibodies [29,30] to achieve quantitative measurements of intracellular endogenous fluorescent analytes or label captured nonfluorescent analytes from single cells with a fluorescent reporter in vitro. However, these fluorescent-based approaches suffer from several pitfalls. First, these strategies are often limited by low sensitivity when targeting low-abundance molecules. Second, the limited availability of endogenous fluorescent analytes in single cells restricts their broader applications.

To overcome these drawbacks, a wide range of functionalization approaches has been developed. Among them, the most popular method involves modifying the surface of the glass pipette to exhibit unique optical or electrochemical characteristics. For example, a metal interface can be introduced to the glass pipette to form a plasmonic glass pipette. Typically, chemical reduction reactions are used to uniformly coat a nanometer-thick layer of gold on the surface of glass pipettes (Figure 2b) [31]. Additionally, noble metal nanoparticle-coated glass pipettes can be fabricated via electrostatic interactions. For instance, gold nanoparticles [32] and gold nanostars [33] have been used for this purpose. A representative example of a gold nanostar-functionalized glass nanopipette is shown in Figure 2c. These plasmonic single-cell endoscopes can be further adapted for the use of advanced detection techniques, such as Raman spectroscopy [31], which provides single-molecule sensitivity for the detection of low-copy-number molecules in single cells.

*AFM tips.* Traditionally, AFM is an invaluable imaging tool capable of scanning cell surfaces using a pyramidal tip mounted on a flexible cantilever. As the AFM tips scan across the cell surface, interaction forces between the tip and the sample cause the cantilever to deflect. This deflection is monitored by a laser beam. In this way, AFM serves as a powerful tool for assessing cell membrane properties, such as elasticity and viscosity [37,38,39]. However, to date, there have been few reports on the detection of intracellular biomolecules using AFM-based force measurements.

Based on the AFM technique, a large volume of published studies has introduced a novel ultrathin nanoneedle, fabricated by using a focused ion beam (FIB) to etch a pyramidal silicon AFM tip [22]. In this system, the diameter of the AFM tip is around 200 nm; the sharpened AFM tip has higher subcellular resolution, which can not only access the cytosol but also penetrate through the nuclear membrane without causing chromosomal DNA damage or apoptosis. Additionally, by measuring the specific mechanical interactions between the antibody-modified tip and the intracellular components, the antibody-functionalized sharp AFM tip can be used for cell screening [23,24]. In addition, fluidic force microscopy (FluidFM), which combines a conventional AFM with microchannel cantilevers connected to a pressure-controlled fluidic circuit, is able to sample intracellular liquid locally [40,41]. A representative FluidFM-based endoscope with an opening size of 400 nm is shown in Figure 2d. In this system, by applying a negative pressure to a hollow pyramidal tip, cell membranes can be controllably penetrated while live intracellular fractions with subcellular spatiotemporal information can be selectively withdrawn. The volumes harvested range between 0.1 and 0.7 pL in a single live cell. It is remarkable that 82% of the cells remain viable after extraction of volumes up to 4.0 pL from cytoplasm [36].

Compared to traditional glass pipettes, the AFM tip-based endoscope functions primarily as a nanoscale sampling tool, enabling the precise and nondestructive longitudinal extraction of ultra-small quantities of intracellular contents [42,43]. After tunable extraction, the obtained cellular contents are amenable to a broader range of analytical techniques, such as fluorescence, enzymatic assays, and PCR-based analysis.

### 2.2. Cylindrical Endoscopes

The primary limitation of conical endoscopes, such as those based on glass pipettes or AFM tips, is their rigid structure and relatively large size compared to cellular dimensions. This restricts their ability to penetrate more than 1 µm into living cells without causing damage and limits their capacity to reach and interrogate deep intracellular organelles. To address these challenges, high-aspect-ratio cylindrical nanostructures, such as nanowires and nanotubes, have been developed. These nanostructures offer significant advantages for intracellular access due to their nanoscale dimensions, high mechanical strength, flexibility, and functionalizable surfaces, making them highly promising for sensing and intracellular delivery applications.

*Nanowire-based endoscopes*. Given the ultra-small dimensions (~50–250 nm) and mechanical flexibility, which could well minimize the damage they inflict on cellular structures and functions, nanowires are promising for interrogating intracellular environments.

**Figure 3:**
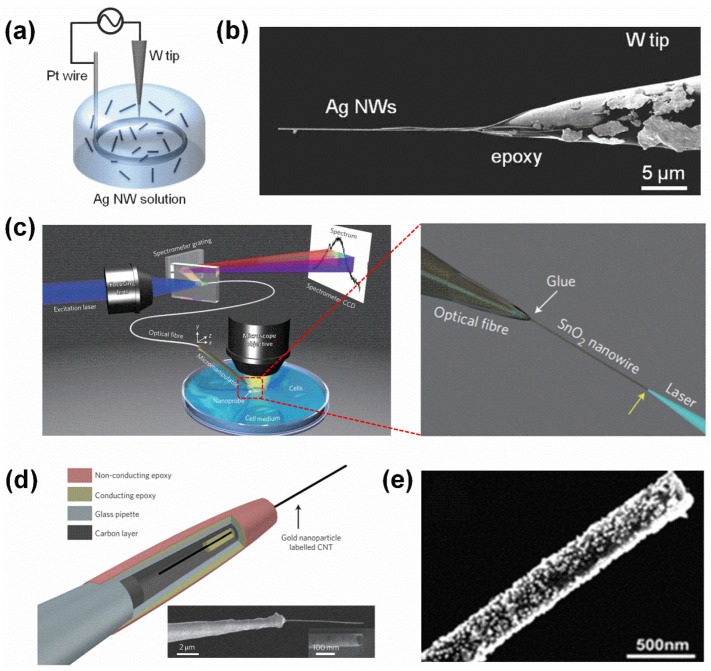
Fabrication and characterization of the cylindrical endoscopes. (**a**,**b**) Schematic of the attachment of AgNWs on an electrochemically etched tungsten tip using the AC dielectrophoresis method. Reproduced with permission [45]. Copyright 2014, John Wiley and Sons. (**c**) Schematic illustration of the nanowire-based endoscope system, with the enlarged one being the three-dimensional schematic showing a blue laser waveguided through a nanowire endoscope constructed by gluing an SnO_2_ nanowire to the tip of a tapered single-mode optical fiber. The yellow arrow points to the nanowire tip where the waveguided light was emitted into free space. Reproduced with permission [18]. Copyright 2012, Springer Nature. (**d**) Schematic of a typical carbon nanotube-based endoscope, showing a multiwalled carbon nanotube attached to the end of a glass pipette, which is coated with a nonconducting epoxy on the outside and a conducting epoxy on the inside. Inserted is an SEM micrograph of an as-assembled endoscope with 50 nm carbon nanotube tips and nanotube tip openings. Epoxy glue seals the glass pipette entrance, and the ends of the nanotube remain open for fluid transfer. Reproduced with permission [19]. Copyright 2011, Springer Nature. (**e**) SEM micrograph of the gold nanoparticle (~20 nm) decorated carbon nanotube tip of a SERS-enabled endoscope using an electrostatic functionalization technique. Reproduced with permission [49]. Copyright 2011, John Wiley and Sons.

A nanowire-based endoscope system usually consists of two parts: one is a cylindrical nanomaterial, such as SnO_2_ [18], ZnO [44], AgNWs [45], AuNWs [46,47], or SiC@C [48], etc., which can be inserted into a single living cell and provides a confined space for intracellular sensing or payload delivery; the other is a supporting platform, such as optical fibers [18], tungsten tips [45], or glass pipettes [19], which can be mounted on a three-dimensional micromanipulator to offer precise control of the position of a nanowire in the single living cell with subcellular resolution. For example, AgNWs are attached to an electrochemically etched tungsten tip using the alternating current (AC) dielectrophoresis method (Figure 3a), and Figure 3b is a typical tungsten tip with 50 nm diameter AgNWs attached and glued with conductive epoxy [45]. As for the selection of supporting platforms, in contrast to tungsten tips and glass pipettes, optical fiber has the advantage of allowing optical information to be easily directed to and extracted from the cells. For example, an optical waveguide endoscope was fabricated by bonding an SnO_2_ nanowire to the tapered tip of an optical fiber (Figure 3c), and when this endoscope was coupled to a target laser before and during deformation by a tungsten needle, it showed the nanowire-based optical endoscope is flexible and robust. The nanowire-based optical endoscope can be optically coupled to either an excitation laser to function as a local light source for subcellular imaging or a spectrometer to collect optical signals [18].

*Carbon nanotube-based endoscopes*. Carbon nanotubes are a significant cylindrical nanomaterial for single-cell endoscopy, owing to their exceptional mechanical strength, tunable diameters, and high electrical conductivity [25].

Similarly to the nanowire-based endoscope, the carbon nanotube-based endoscope also consists of two major sections: a carbon nanotube and a supporting platform. Generally, a multiwalled carbon nanotube, with a length of around 50–60 µm, an outer diameter of 50 nm to 200 nm, and a thickness of 10–25 nm, is fixed to the tip of a conventional glass pipette using a flow-through technique [19], and Figure 3d shows a typical nanotube endoscope, showing a multiwalled carbon nanotube attached to the end of a glass pipette, which is coated with a nonconducting epoxy on the outside and a conducting epoxy on the inside.

When functionalized with various nanoparticles, these nanotube-tipped endoscopes are able to acquire diverse, unique properties. For example, magnetic nanotube endoscopes [50] are produced when a nanotube is filled with superparamagnetic nanoparticles. Through this modified endoscope, atto-liter volumes of fluids can be remotely transferred to and from exact locations. Additionally, to create SERS-active nanotube endoscopes (Figure 3e) [49], an electrostatic functionalization technique is employed to decorate nanotubes with gold nanoparticles. This SERS-active nanotube endoscope exhibits high selectivity and sensitivity, enabling it to detect real-time SERS activity of DNA and other important biomolecules in situ within a single cell in a minimally invasive manner.

### 2.3. Vertically Aligned Arrays

The mentioned single-cell endoscopes all offer high levels of control over cell selection and intracellular extraction volume, yet uniformly come at the expense of throughput. To overcome this deficiency, three-dimensional vertically aligned arrays, consisting of random or ordered arrangements of high-aspect-ratio hollow nanostructures, have emerged as promising tools to sense or monitor the intracellular events by inserting the hollow nanostructures into living cells.

**Figure 4:**
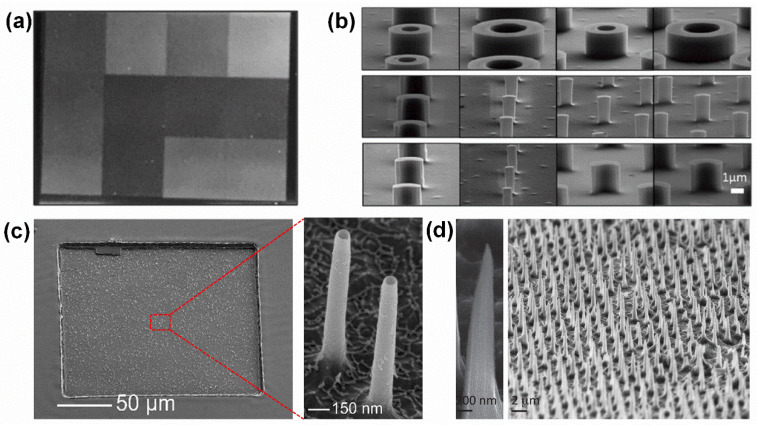
Fabrication and characterization of the vertically aligned arrays. (**a**) Digital image of the silicon platform showing 12 different micropillar arrays fabricated on a single device. Reproduced with permission [52]. Copyright 2016, American Chemical Society. (**b**) High-magnification SEM micrograph of each micropillar in the array. Reproduced with permission [52]. Copyright 2016, American Chemical Society. (**c**) SEM image of a 200 × 200 µm active sampling region and tilted view (45°) SEM images of the 150 nm diameter nanostraw on the arrays. (**d**) Scanning electron micrographs of a uniform array of conical pSi nanoneedles, with <100 nm tip diameter, 600 nm base diameter, 5 µm length, and 2 µm pitch. Reproduced with permission [21]. Copyright 2017, American Chemical Society.

Currently, a variety of materials have been used to fabricate these vertically aligned arrays, such as silica [26,27], alumina [28], and indium arsenide [51]. For example, a new microfabricated platform consisting of rigid silicon micropillar arrays of various shapes, sizes, and configurations fabricated side by side on a silicon platform (Figure 4a), and the outer diameters of the micropillars were measured to range from 0.7 to 5.5 μm with the height at 2 μm, and in all arrays, a constant 5 μm side-to-side spacing was maintained between neighboring micropillars, resulting in comparable free flat areas between the micropillars irrespective of their sizes (Figure 4b) [52]. Moreover, a nondestructive nanostraw extraction (NEX) process is based on diffusively sampling material from inside the cells using a nanostraw (NS)-embedded substrate, usually a polymer membrane, and a defined 200 × 200 μm active region of 150 nm diameter hollow cylinders hollow NS extending through the polymer and producing from the surface (Figure 4c) [21]. These high-aspect-ratio hollow nanostructures on the above vertically aligned arrays can build channels between intracellular and extracellular components across cell membranes, through which the cytoplasmic fractions could be pumped out and transferred to conventional methods, such as fluorescence, enzymatic assays (ELISA), and quantitative real-time PCR.

It is also found that two pivotal factors that influence single-cell endoscopy are the dimension and arrangement of the arrays, especially on cell membrane penetration and intracellular sampling efficiency. For example, studies have shown that small-diameter nanowires, typically around 100 nm in diameter (a typical uniform array of conical nanoneedles is illustrated in Figure 4d), are capable of penetrating cells. In contrast, cell penetration is unlikely to occur with larger nanostructure arrays, where the diameter exceeds 250 nm [21]. As for acquiring appropriate vertically aligned arrays-based endoscopes to precisely control the position and spacing of the arrays, various techniques have been utilized, such as e-beam lithography (EBL) [53], nanoimprinting [54], etc. Compared with other endoscopes, vertically aligned arrays are advantageous for performing high-throughput single-cell experiments; more to the point, they can monitor intracellular events over a relatively long-term period.

## 3. Biomedical Applications

Specific markers, such as nucleic acids (DNA and RNA), proteins, and trace elements, are crucial for understanding the molecular and cellular mechanisms of related diseases. However, detecting disease-related markers in single living cells remains challenging due to their low abundance [11]. Thanks to the distinguished virtues of the various endoscopes mentioned, such as flexible live-cell manipulation, high specificity, and single-molecule sensitivity, it is now possible to directly extract native intracellular biomolecules without compromising cell viability. When further combined with advanced detection techniques, such as Raman spectroscopy and electrochemical detection, these endoscopes facilitate the sensing of a wide range of significant intracellular biomolecular events, thereby providing deeper insights into disease mechanisms and potential therapeutic targets.

### 3.1. Nucleic Acids

**Figure 5:**
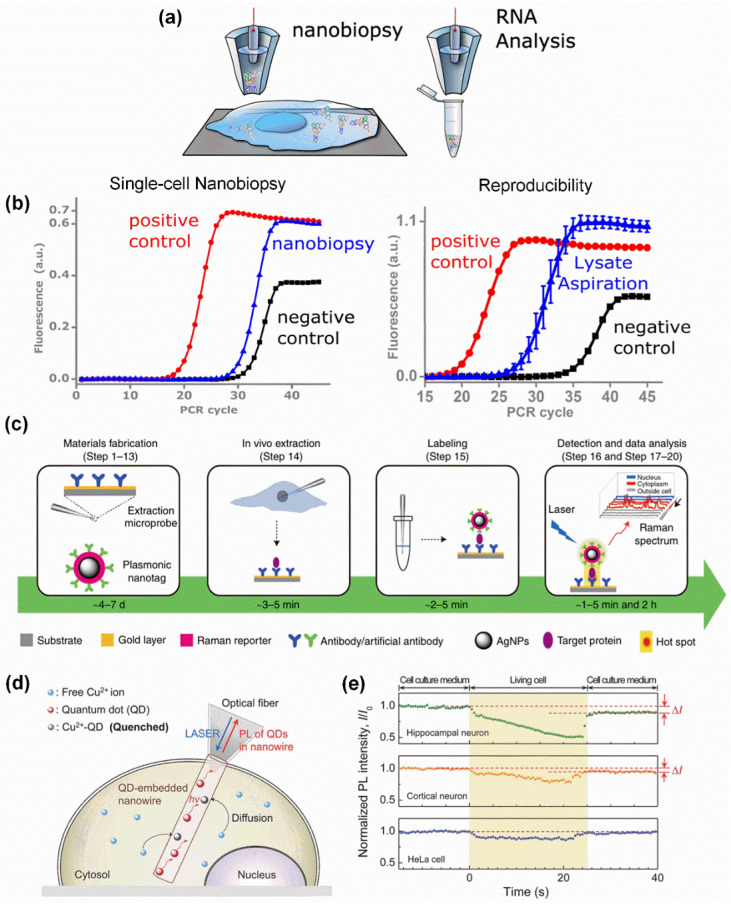
**Intracellular biomolecular sensing by single-cell endoscopy technique.** (**a**) RNA analysis based on electrowetting within a nanopipette. (**b**) Postbiopsy analysis via qPCR targeting GFP RNA from HeLa cells showing a nanobiopsy taken from a single cell and the reproducibility of nanobiopsy protocols. Reproduced with permission [55]. Copyright 2014, American Chemical Society. (**c**) Quantitative measurement of low-copy-number proteins in single living cells by single-cell plasmonic immune sandwich assay (scPISA). Reproduced with permission [35]. Copyright 2021, Springer Nature. (**d**) Schematic illustration of quantitative probing of Cu^2+^ ions naturally present in a living cell, based on an active nanowire-based waveguide probe. (**e**) Normalized PL intensity (I/I_0_) with time for single living hippocampal (green dots) and cortical neurons (orange dots) and a Hela cell (red dots) as a reference. Reproduced with permission [57]. Copyright 2016, John Wiley and Sons.

Specific gene expression analysis in single living cells has the potential to become a crucial technique in cell biology. However, conventional methods have been unable to detect nucleic acids in living cells without killing or destroying them. The advent of single-cell endoscope techniques has effectively overcome these limitations in the analysis of nucleic acids, including mRNA [55], microRNAs [34], tRNA [56], etc. For example, a novel platform for genomic analysis based on electrowetting within a nanopipette (Figure 5a) has been developed. This platform can isolate small subpopulations of mitochondria from single living cells and further quantify mutant mitochondrial genomes using high-throughput sequencing technology. The results obtained from single-cell experiments were highly consistent with those from cell lysate-based measurements, providing a foundation for dynamic subcellular genomic analysis (Figure 5b) [55]. Moreover, a platform based on fluidic force microscopy (fluidFM) has enabled the quantitative extraction of cytoplasmic and nucleoplasmic fractions from single living cells. The withdrawn cell contents can then be delivered for quantitative PCR (qPCR) to detect mRNA levels. This pioneering work in single-cell transcript analysis helps researchers explore the relationship between cell growth and development, tumor cell metastasis, and cellular characteristics [36].

### 3.2. Proteins

Despite impressive advances in the analysis of nucleic acids in single living cells, the investigation of proteins in single living cells has relatively lagged behind, primarily because proteins cannot be amplified in the same way as nucleic acids. As essential constituents of cells, proteins play vital roles in cellular behaviors and functions. In particular, low-copy-number proteins are indispensable in cell functions, such as signaling and gene expression. Increasing evidence has confirmed that abnormal protein expression is associated with various human diseases, including cancer [58,59].

The advent of single-cell endoscopy techniques has provided a valuable strategy to analyze low-copy-number proteins in single living cells because it can flexibly combine with some advanced detection techniques, such as Raman spectroscopy [35,60,61], allowing detection limits as low as single-molecule sensitivity. Specifically, a creative single-cell plasmonic immune sandwich assay (scPISA) (Figure 5c) [35], which relies on an antibody-functionalized gold-based plasmonic glass pipette and antibody-modified silver-based plasmonic labeling nanotag, has been developed for the quantitative measurement of various low-copy-number cytoplasmic and nuclear proteins in single living cells with subcellular resolution, including survivin, alkaline phosphatase (ALP), capase-3, cytochrome C (Cyt C), etc. [20,35,60,61] The unique mode of in vivo extraction and in vitro labeling makes it possible to detect intracellular biomolecules with high sensitivity (~6 protein molecules) [20].

### 3.3. Ions

Besides the nucleic acids and proteins, some ions also act as essential components in a host of fundamental biological processes, such as copper ions (Cu^2+^), calcium ions (Ca^2+^), trivalent iron (Fe^3+^), etc. For example, Cu^2+^ is closely related to severe neurodegenerative diseases, namely Alzheimer’s disease and Parkinson’s disease. Accurate detection of naturally occurring Cu^2+^ in living cells is crucial for understanding its role in neurological diseases. However, existing methods have limitations, particularly as fluorescence microscopy cannot achieve quantitative detection of Cu^2+^ within these cells. However, X-ray fluorescence microscopy is used for chemically or freeze-fixed cells and cannot detect natural live cells.

A probe was designed by embedding CdSe/ZnS quantum dots (QDs) into polycaprolactone (PCL) nanowires to serve as active nanowire waveguides for the quantitative detection of Cu^2+^ ions naturally present in single living cells (Figure 5d). The intracellular Cu^2+^ ion concentration was quantified through direct monitoring of photoluminescence quenching during the insertion of the nanowire in a living neuron. The high biocompatibility, stability, selectivity, and quantum yield of the probe for Cu^2+^ ions enabled the measurement of an intracellular Cu^2+^ ion concentration of 3.34 ± 1.04 × 10^−6^ M (mean ± s.e.m.) in single hippocampal neurons (Figure 5e) [57]. As a second messenger, Ca^2+^ can play an important role in various cellular processes. Detecting Ca^2+^ in cells is of great significance for understanding cellular function, disease research and treatment, and drug development. A novel nanoprobe that combines SERS and fluorescence techniques can be used for the detection of Ca^2+^ in cells. This probe is composed of Au nanostars (AuNSs) and DNAzymes. AuNSs serve to quench fluorescence while enhancing Raman signals, while DNAzymes can specifically recognize Ca^2+^ and trigger fluorescence recovery and SERS signal attenuation, thereby achieving high selectivity and sensitivity detection of Ca^2+^ in live cells [62]. Detecting intracellular Fe^3+^ is of great significance for the early diagnosis and treatment of anemia, organ dysfunction, and various diseases. A nanoprobe based on surface-enhanced Raman scattering (SERS) technology can be used for the quantitative detection of Fe^3+^ and heme proteins in single cells. The probe is coated with a thin gold film at the tip of the glass capillary to enhance SERS and is modified with organic cyanide (MBN). The probe has high sensitivity and selectivity for Fe^3+^ and heme proteins and can achieve quantitative detection of them. It can also be regenerated repeatedly after EDTA treatment [63].

### 3.4. Cellular Microenvironment Monitoring

**Figure 6:**
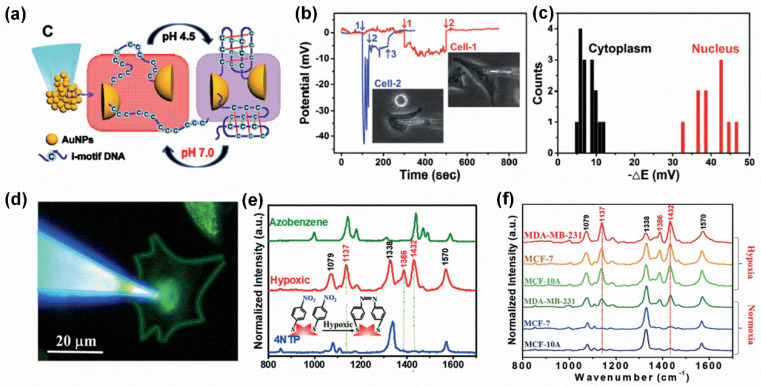
**Single-cell endoscopy technique in cellular microenvironment monitoring.** (**a**) Local pH probing. (**b**) Potential time curves of the GPS when it penetrated through different cells. (**c**) The _Δ*E* statistical diagram of the GPS in different cells. Reproduced with permission [64]. Copyright 2018, John Wiley and Sons. (**d**) Intracellular hypoxia sensing based on a Raman lancet with a 4NTP redox marker. (**e**) Raman spectra of 4-NTP on a Raman lancet exposed to hypoxic cells. (**f**) SERS spectra in the three different cells under normoxia and hypoxia conditions. Reproduced with permission [33]. Copyright 2019, John Wiley and Sons.

Aside from the above biomarkers, single-cell endoscopy techniques can also perform intracellular environment monitoring, such as pH [64,65] and hypoxia [33], which are two key factors for cells as they influence cell functions, behaviors, and pathological conditions. For instance, a glass nanopipette designed with a pH-sensitive gold porous sphere (GPS) on the tip, with the help of i-motif DNA thiolated at both ends as linker molecules, and the size of the formed nanopores can be tuned by the folded-unfold conformational change in i-motif DNA upon pH adjustment from 4.5 to 7.0 (Figure 6a). Moreover, the successful applications of GPS in multiparameter pH probing in different regions of single HeLa cells suggested that the combination of plasmonic metal with nanopores and glass nanopipette provides a promising nanodevice in single-cell analysis (Figure 6b,c) [64].

In addition to intracellular pH, the amount of gas level is another important factor affecting the cellular environment, such as hypoxia, an insufficient oxygen supply state, which is commonly found in tumor microenvironments, and accurate measurements of hypoxic levels in cells may predict the proper response to treatment and disease outcome in cancer patients because hypoxia is usually associated with a poor prognosis across multiple tumor types. For example, a potent intracellular hypoxia-sensing Raman probe was designed, which functionalized a nanopipette tip with multiple sharp-edged gold nanostars through electrostatic interactions and was used to quantify oxygen levels in hypoxic single cells (Figure 6d). Distinct Raman spectral changes for the nitro-(NO_2_) functional group of the redox marker 4-nitrothiophenol (4-NTP) on the surface of the Raman probe can be quantified according to the intracellular oxygen (O_2_) content, ranging from 1% to 10% (Figure 6e). Through this method, the SERS spectrum of noncancerous cell lines (MCF-10A), weakly metastatic cancer cell lines (MCF-7), and invasive human breast cancer cell lines (MDA-MB-231) can be precisely distinguished under both normoxic and hypoxic conditions (Figure 6f) [33]. Additionally, intracellular ROS and RNS expression levels in living cells were also measured with single-cell endoscopy [66].

## 4. Conclusions and Perspectives

Single-cell endoscopy provides a confined space for discovering substances and activities with high resolution, specificity, and sensitivity in single living cells. It has attracted increasing attention in recent years. However, despite its numerous advantages, single-cell endoscopy also faces some challenges. One of the most significant limitations is the relatively low throughput, which restricts its application in large-scale screening and high-throughput analysis. To address this issue, several strategies could be explored. For example, the development of microfluidic-based single-cell endoscopy systems could potentially increase the throughput by enabling parallel processing of multiple cells. Additionally, integrating single-cell endoscopy with automated imaging and data analysis platforms may further enhance its efficiency and applicability. In addition, the combination of single-cell endoscopy with other advanced detection technologies such as Raman spectroscopy, electrochemical detection, etc., will greatly expand the practical applications of single-cell endoscopy, enabling it to have a wider range of applications in research fields such as disease diagnosis, drug delivery, and molecular biology.

In summary, while single-cell endoscopy holds great promise for advancing our understanding of cellular heterogeneity and disease mechanisms, efforts should be made to overcome its current limitations. By focusing on improving throughput and combining it with other cutting-edge technologies, we foresee this confined single-cell endoscopy technique will gain rapid development and find more promising applications in disease mechanism research and early screening, drug development, and cell exploration.

## Data Availability

No primary research results, software, or code have been included, and no new data were generated or analyzed as part of this review.
